# Clinicopathological implications of Tiam1 overexpression in invasive ductal carcinoma of the breast

**DOI:** 10.1186/s12885-016-2724-0

**Published:** 2016-08-25

**Authors:** Zhenling Li, Qixiang Liu, Junjie Piao, Fenjian Hua, Jing Wang, Guang Jin, Zhenhua Lin, Yan Zhang

**Affiliations:** 1 Department of Pathology & Cancer Research Center, Yanbian University Medical College, Yanji, 133002 China; 2 Department of Breast Surgery, the Second Hospital of Jilin University, Changchun, 130041 China

**Keywords:** Breast cancer, Tiam1, Immunohistochemistry, Prognosis, Survival analysis

## Abstract

**Background:**

T-lymphoma invasion and metastasis-inducing protein 1 (Tiam1) has been implicated in tumor occurrence and progression. Recent studies have shown that high expression levels of Tiam1 protein appear to be associated with the progression of numerous human tumors. This study attempted to explore the role of Tiam1 protein in tumor progression and the prognostic evaluation of breast cancer.

**Methods:**

The localization of the Tiam1 protein was determined in the MDA-MB-231 breast cancer cell line using immunofluorescence (IF) staining. In addition, a total of 283 breast tissue samples, including 153 breast cancer tissues, 67 ductal carcinoma in situ (DCIS) and 63 adjacent non-tumor breast tissues, were analyzed by immunohistochemical (IHC) staining of the Tiam1 protein. The correlation between Tiam1 expression and clinicopathological characteristics was evaluated by Chi-square test and Fisher’s exact tests. Disease-free survival (DFS) and 10-year overall survival (OS) rates were calculated by the Kaplan-Meier method. Additionally, univariate and multivariate analyses were performed by the Cox proportional hazards regression models.

**Results:**

Tiam1 protein showed a mainly cytoplasmic staining pattern in breast cancer cells; however, nuclear staining was also observed. Tiam1 protein expression was significantly higher in breast cancers (42.5 %, 65/153) and DCIS (40.3 %, 27/67) than in adjacent non-tumor tissues (12.7 %, 8/63). In addition, Tiam1 associated with tumor stage and Ki-67 expression, but negatively correlated with receptor tyrosine-protein kinase erbB-2 (Her2) expression. Moreover, survival analyses showed that DFS and 10-year OS rates were significantly lower in breast cancer patients with high Tiam1 expression than those with low Tiam1 expression. Univariate analysis suggested that molecular types, clinical stage, Her2 expression levels and Tiam1 expression levels were also significantly associated with DFS and 10-year OS rates of breast cancer patients. Furthermore, multivariate analysis suggested that Tiam1 expression is a significant independent prognostic factor along with tumor stage in patients with breast cancer.

**Conclusions:**

Tiam1 expression is frequently up-regulated in breast cancer. Tiam1 expression correlated with clinicopathological parameters, suggesting that it may be a useful prognostic biomarker and potential therapeutic target for patients with breast cancer.

## Background

Breast cancer is one of the most common tumors in women worldwide, accounting for approximately 29 % of all new cancer cases among women, and it is the most common cause of cancer death in women. Specifically, more than 226,870 new cases and 39,510 deaths are reported each year worldwide [1]. Ductal carcinoma in situ (DCIS) is defined as local disease involving the proliferation of abnormal epithelial cells that have not crossed the basement membrane and invaded the stroma, and is considered to be a non-obligate precursor to invasive breast cancer [2]. Moreover, invasive breast cancer includes at least four major molecular subtypes, which differ by their expression of estrogen receptor alpha (ER), progesterone receptor (PR), receptor tyrosine-protein kinase erbB-2 (Her2), and the proliferative status of the tumor [3, 4]. The levels of ER, PR and Her2 expression are closely related to breast cancer, and have been used for predicting the outcomes and response to breast cancer therapy. Currently, although therapeutic strategies have improved, existing prognosis factors have failed to provide the necessary precision needed for making therapeutic decisions.

T-lymphoma invasion and metastasis-inducing factor 1 (Tiam1) was first identified as an invasion and metastasis-related gene by Habets et al. [5] in mice with aggressive T-cell lymphoma. Tiam1 is a guanine nucleotide exchange factor (GEF) and regulates guanosine triphosphatase to facilitate the exchange of guanosine diphosphate for guanosine triphosphate. Tiam1 expression plays an important role in tumor progression and metastasis by activating Rho-like GTPases, specifically the Tiam1-Rac pathway, which participates in cell migration, invasion and metastasis [6, 7]. Moreover, the increased expression of Tiam1 has been reported in a variety of tumor types, such as colorectal carcinoma [8], hepatocellular carcinoma [9], prostate carcinoma [10], lung carcinoma [11] and squamous-cell carcinoma of the head and neck [12]. Overexpression of Tiam1 protein participates in many processes underpinning tumor progression [7, 13], including apoptosis, lymphangiogenesis, invasion and migration. All these findings indicate that Tiam1 expression might be a new and independent predictor of prognosis in various tumors, and Tiam1 may be a potential target for tumor therapy. However, there are relatively few published reports evaluating the role of Tiam1 protein expression in breast cancer.

Therefore, in this study, a total of 153 breast cancer samples, 67 DCIS and 63 adjacent non-tumor breast tissues were evaluated for the expression of Tiam1 by immunohistochemical (IHC) staining. Herein, we report that the expression of Tiam1 is important in the tumorigenesis of breast cancer and could serve as a prognostic marker.

## Methods

### Ethics statement

This research complied with the Helsinki Declaration and was approved by the Human Ethics Committee and the Research Ethics Committee of Yanbian University Medical College. Patients were informed that the resected specimens were stored by the hospital and potentially used for scientific research, and that their privacy would be maintained. Follow-up survival data were collected retrospectively through medical record analyses.

### Clinical samples

Total 283 tissue samples were used for this study, including 153 were breast cancer samples, 67 DCIS and 63 adjacent non-tumor breast tissues. All of these tissues were collected from Shanghai Outdo Biotech Co. Ltd. (Outdo Biotech) and Tissue Bank of Yanbian University Medical College. All tissues were routinely fixed in 10 % buffered formalin and embedded in paraffin blocks. The study protocol was approved by the institutional review board of Yanbian University Medical College. The pathological parameters, including age, menopausal status, molecular type, tumor size, LN metastasis, ER expression, PR expression, Her2 expression and Ki-67 expression were carefully reviewed in all 153 breast cancer cases. All cases were confirmed with breast cancer by pathological examination. Clinicopathological classification and staging were assessed according to the American Joint Committee on Cancer (AJCC) criteria. Clinical information of the samples is summarized in Table 2.

### Cell culture

MDA-MB-231 human breast cancer cells were supplied by the Cancer Research Center of Yanbian University. The cells were plated onto cell culture dishes, and cultured in 10 % fetal bovine serum L-15 medium. And then, the cells maintained at 37 °C in a humidified incubator in an atmosphere of 5 % CO_2_.

### Immunofluorescence (IF) staining of the Tiam1 protein in MDA-MB-231 breast cancer cells

The breast cancer cell line, MDA-MB-231, was grown on coverslips to 70 % confluence and then the cells were fixed in 4 % paraformaldehyde in PBS for 10 min at RT, and permeabilized with 0.5 % TritonX-100 for 10 min. Blocking was performed with 3 % bovine serum albumin fraction V (A8020, Solarbio, Beijing, China) for 1 h at RT. After washing with phosphate-buffered saline (PBS), cells were incubated with anti-rabbit Tiam1 (1:500, Santa Cruz Biotechnology) at 4 °C overnight, followed by incubation with Alexa Fluor® 488 goat anti-rabbit IgG (H + C) (A11008, Invitrogen, USA) respectively, for 1 h at RT. After washing with PBS, cells were counterstained with 4’,6-diamidino-2-phenylindole (DAPI) (C1006, Beyotime, Shanghai, China) and the coverslips were mounted with Antifade Mounting Medium (P0126, Beyotime, Shanghai, China). Finally, the immunofluorescence signals were visualized and recorded by Leica SP5II confocal microscope.

### Immunohistochemistry (IHC) for Tiam1 protein in paraffin-embedded tissues

IHC analysis was performed using the DAKO LSAB kit (DAKO A/S, Glostrup, Denmark). Briefly, to eliminate endogenous peroxidase activity, 4 μm thick tissue sections were deparaffinized, rehydrated and incubated with 3 % H_2_O_2_ in methanol for 15 min at RT. The antigen was retrieved at 95 °C for 20 min by placing the slides in 0.01 M sodium citrate buffer (pH 6.0). The slides were then incubated with Tiam1 antibody (1:600, Santa Cruz Biotechnology, USA) at 4 °C overnight. After incubation with biotinylated secondary antibody at RT for 30 min, the slides were incubated with streptavidin-peroxidase complex at RT for 30 min. IHC staining was developed by using 3,3′-diaminobenzidine, and Mayer’s hematoxylin was used for counterstaining. In addition, the positive tissue sections were processed with omitting of the primary antibody as negative controls.

### Evaluation of IHC staining

All specimens were examined by two investigators (Jin T & Lin Z) who did not possess knowledge of the clinical data. In case of discrepancies, a final score was established by reassessment on a double-headed microscope. Briefly, the IHC staining for Tiam1 was semi-quantitatively scored as ‘−’ (negative, no or less than 5 % positive cells), ‘+’ (6–25 % positive cells), ‘++’ (26–75 % positive cells), and ‘+++’ (more than 75 % positive cells, considered as strongly positive).

### Statistical analysis

Statistical analyses were performed using the SPSS software program for windows, version 17.0 (SPSS, Inc., Chicago, IL, USA), and the JMP software program for Mac, version 10.0.0 (SAS Institute Inc., Cary, NC, USA). Correlation between Tiam1 expression and clinicopathological characteristics were evaluated by Chi-square test and Fisher’s exact tests. The survival rates after tumor removal were calculated by the Kaplan-Meier method, and differences in survival curves were analyzed by the Log-rank tests. Multivariate survival analysis was performed on all the significant characteristics measured by univariate survival analysis through the Cox proportional hazard regression model. *P*-values less than 0.05 were considered statistically significant.

## Results

### Tiam1 protein expression is increased in breast cancers

**Fig. 1:**
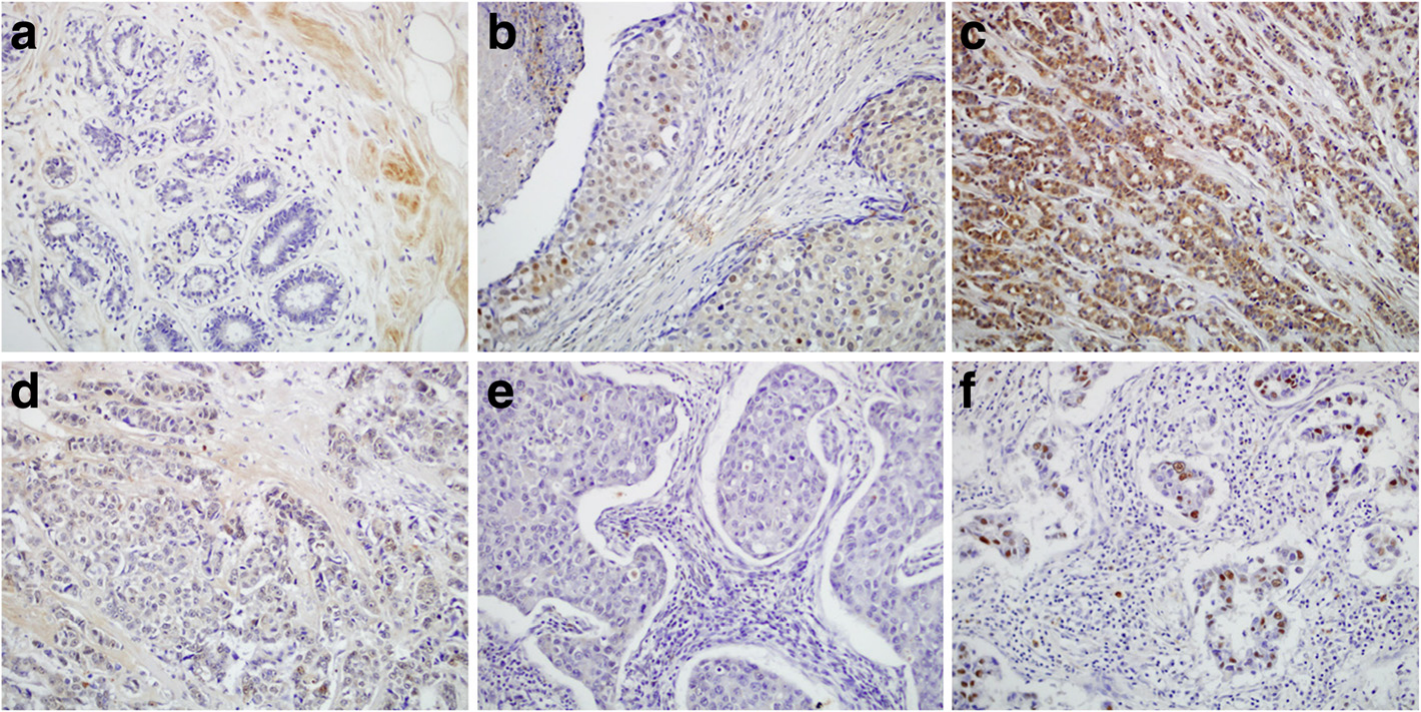
IHC staining of Tiam1 protein in breast cancer tissues. **a** Lack of Tiam1 protein staining in normal breast tissues. **b** Tiam1 protein was showed weakly positive in DCIS tissues. **c** Diffuse and strong positive expression of Tiam1 protein staining in breast cancer cells with LN metastasis (Luminal B). **d** Tiam1 was weakly positive in breast cancers with LN metastasis (Luminal B). **e** Lack of Tiam1 protein staining in breast cancers without metastasis (triple negative). **f** Tiam1 protein staining localized some nuclear staining was also observed in the metastatic breast cancers with lymphatic vessel invasion (triple negative). (Original magnification, A-F: ×200)

To determine the expression levels of Tiam1 in breast cancer patients, we performed IHC. The staining pattern of Tiam1 protein was primarily cytoplasmicin breast cancers, but nuclear staining was also observed (Fig. 1). The proportion of samples with high Tiam1 expression was 76.5 % (117/153) in breast cancer; it was significantly higher than in DCIS (51.1 %, 35/67) or adjacent non-tumor tissues (28.6 %, 18/63). Similarly, the proportion of samples with strongly positive Tiam1 expression was 42.5 % (65/153) in breast cancer and40.3 % (27/67) in DCIS, which were significantly higher than that of adjacent non-tumor tissues (12.7 %, 8/63) (*P* < 0.01, respectively) (Table 1).

**Table 1:** Tiam1 expression in breast cancers

Diagnosis	No. of cases	Positive cases				Positive cases rates	Strongly positive rates
		−	+	++	+++		
Breast cancers	153	36	52	41	24	76.5 %**	42.5 %**
DCIS	67	32	8	14	13	51.1 %**	40.3 %**
Adjacent non-tumor	63	45	10	8	0	28.6 %	12.7 %

*DCIS* ductal carcinoma *in situ*

Positive rate: percentage of positive cases with + ~+++ staining score

Strongly positive rate: percentage of positive cases with ++ and +++ staining score

** *p* < 0.01 compared with non-tumor tissues

**Fig. 2:**
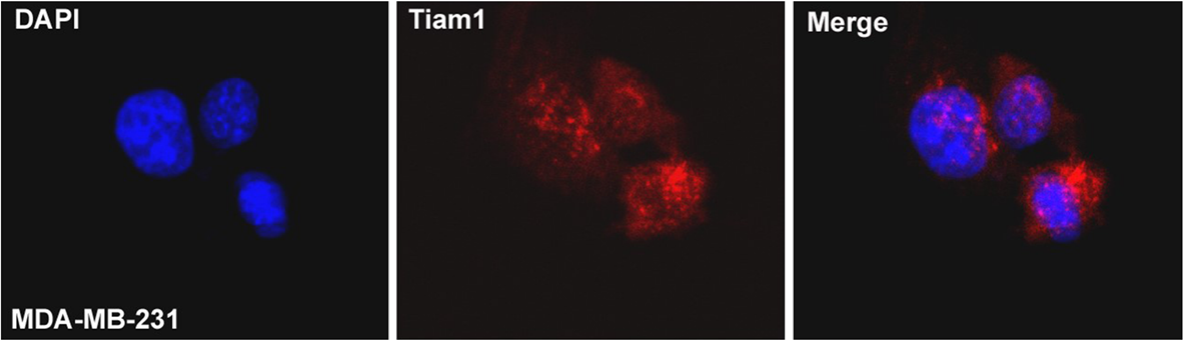
Immunofluorescence staining of the Tiam1 protein in MDA-MB-231 breast cancer cells. The Tiam1 protein was mainly located in the cytoplasm of MDA-MB-231 breast cancer cells, and nuclear staining pattern was also observed (red indicates Tiam1 staining, blue indicates DAPI)

The subcellular localization of Tiam1 protein was further explored by performing IF staining for Tiam1 protein in MDA-MB-231 breast cancer cells. The staining results clearly showed that Tiam1 protein is mainly located in the cytoplasm of MDA-MB-231 breast cancer cells, while some nuclear staining was also observed (Fig. 2).

### Clinicopathological significance of Tiam1 overexpression in patients with breast cancer

**Fig. 3:**
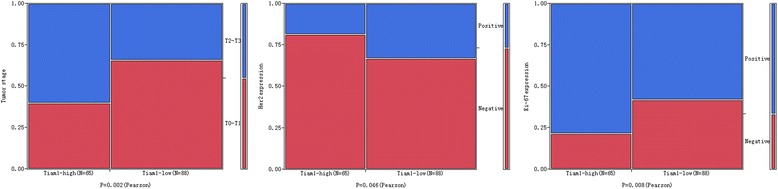
Relationship between Tiam1 expression and clinicopathological significance of breast cancer. The expression level of Tiam1 protein was significantly related to tumor stage (*P* = 0.002), Her2 expression (*P* = 0.046) and Ki-67 expression (*P* = 0.008)

To evaluate the role of Tiam1 protein in breast cancer progression, the relationship between the overexpression of Tiam1 protein and the clinicopathological parameters of patients were analyzed. According to the clinical parameters of the breast cancer patients listed in Table 2, Tiam1 overexpression was not related to patient age, menopausal status, molecular type, lymph node (LN) metastasis, ER or PR expression levels (*P* > 0.05, respectively). However, for tumor stage, we found strongly positive immunostaining (56.5 %, 39/69) in breast cancer patients with T2-T3 (>5.0 cm) tumor size, but only 31 % (26/84) in cases with T0-T1 (≤5.0 cm) tumor size (*P* = 0.002). In addition, Tiam1 was significantly higher in breast cancers with high Ki-67 expression (50.0 %, 51/102) than in cases with low Ki-67 expression (27.5 %, 14/51) (*P* = 0.008). Interestingly, the strongly positive rate of Tiam1 expression was 47.3 % (53/112) in breast cancers with HER2 negative expression, but only 29.3 % (12/41) in case with HER2 positive expression (*P* = 0.046) (Fig. 3).

**Table 2:** Correlation between Tiam1 expression and the clinicopathological features of breast cancer

Variables	No. of cases	Tiam1 strongly positive cases (%)	*χ* ^*2*^	*P* value
Age			0.186	0.666
≥ 50	84	37 (44.0 %)		
< 50	69	28 (40.6 %)		
Menopausal status			1.981	0.159
Premenopausal	63	31 (49.2 %)		
Postmenopausal	90	34 (37.8 %)		
Molecular type			5.142	0.162
Luminal A	39	21 (53.8 %)		
Luminal B	46	21 (45.7 %)		
TNBC	39	15 (35.9 %)		
Her2+	29	8 (27.6 %)		
Tumor stage			10.136	0.002**
T0-T1	84	26 (31.0 %)		
T2-T3	69	39 (56.5 %)		
LN metastasis			0.364	0.546
Absent	57	26 (45.6 %)		
Presence	96	39 (40.6 %)		
ER			0.453	0.501
Positive	87	39 (44.8 %)		
Negative	66	26 (39.4 %)		
PR			0.489	0.484
Positive	78	31 (39.7 %)		
Negative	75	34 (45.3 %)		
Her2			4.003	0.046*
Positive	41	12 (29.3 %)		
Negative	112	53 (47.3 %)		
Ki-67			7.075	0.008**
Positive	102	51 (50.0 %)		
Negative	51	14 (27.5 %)		

* *p* < 0.05, ** *p* < 0.01

### Tiam1 overexpression predicts poor survival rates in patients with breast cancer by the Kaplan-Meier method

**Fig. 4:**
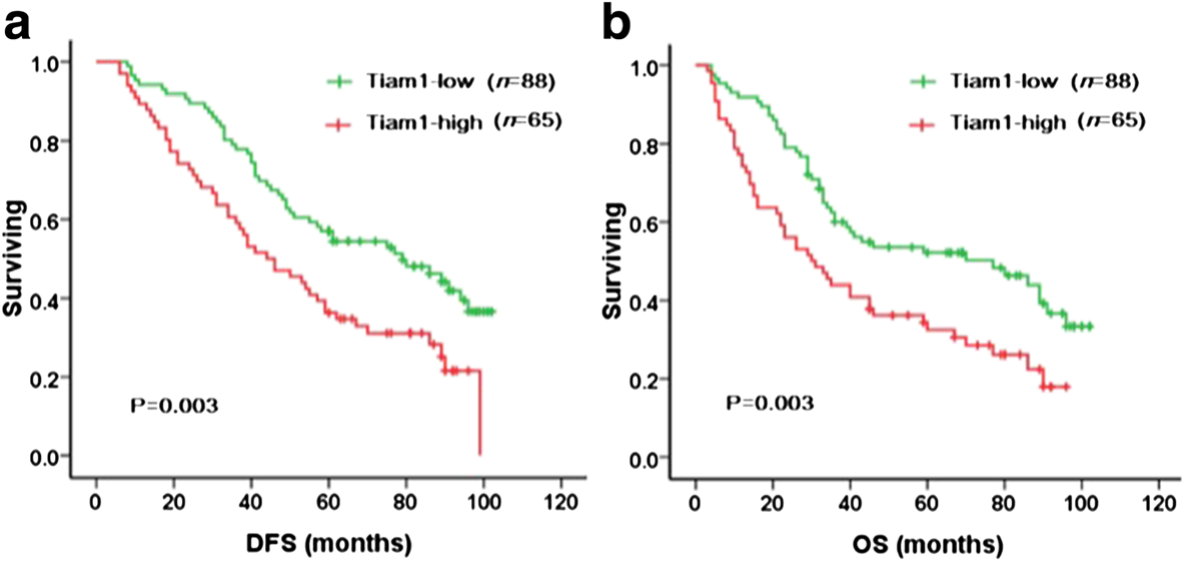
Kaplan-Meier analysis of breast cancer patient survival rates in relation to Tiam1 protein expression. Disease-free survival (**a**) and 10-year overall survival (**b**) rates of patients with high (red, *n* = 65) and low (green, *n* = 88) Tiam1 expression

**Fig. 5:**
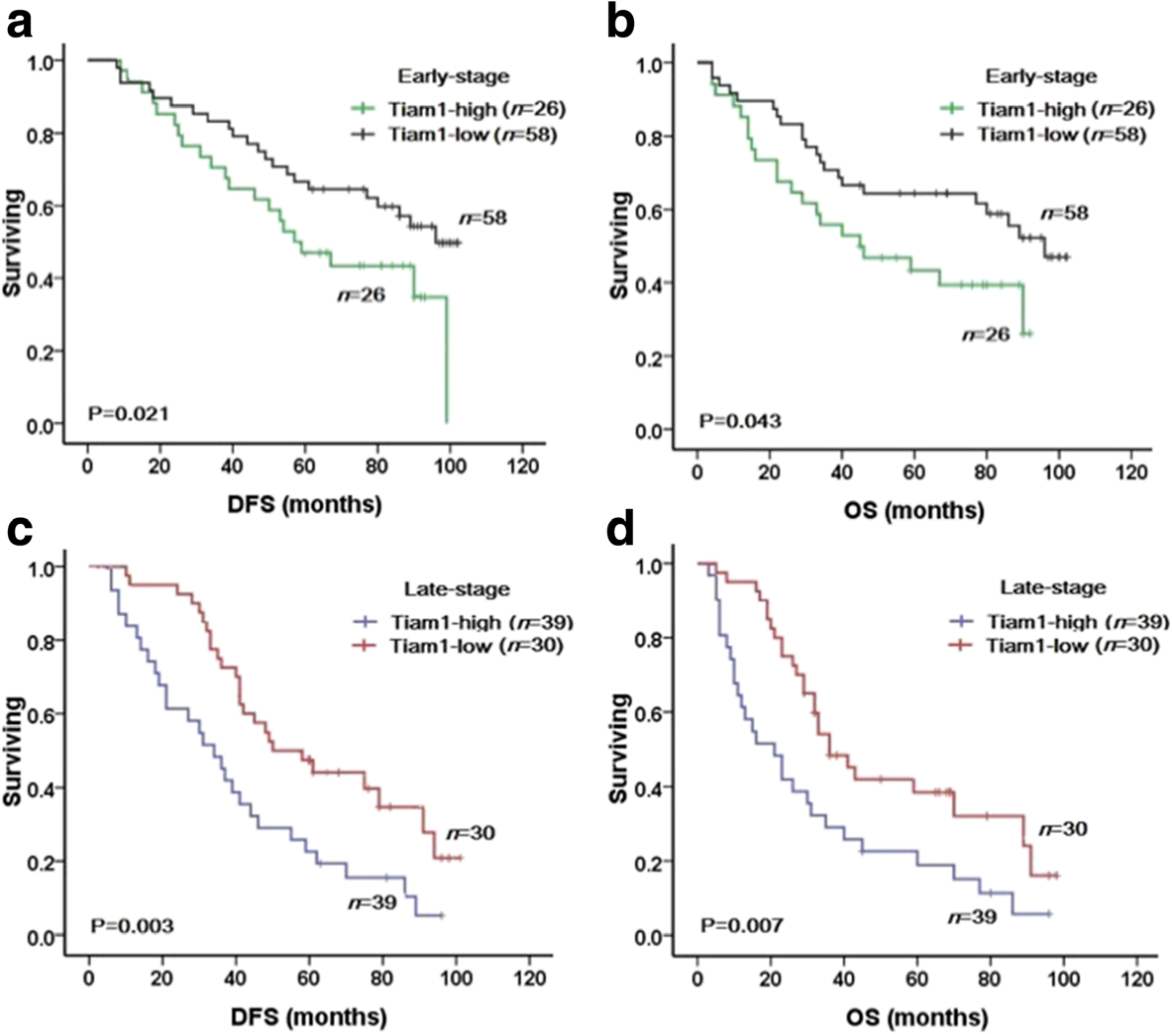
Kaplan-Meier analyses of survival rates in patients with high- or low-level Tiam1 expression and with early- or late-stage breast cancer. DFS (**a**) and OS (**b**) rates were assessed in patients with early-stage breast cancer concomitant with either high- (green, *n* = 26) or low-level (black, *n* = 58) Tiam1 expression. DFS (**c**) and OS (**d**) rates were also assessed in patients with late-stage breast cancer concomitant with high- (blue, *n* = 39) or low-level (red, *n* = 30) Tiam1 expression

To further substantiate the importance of Tiam1 overexpression in breast cancer progression, 153 breast cancer cases were analyzed using the Kaplan-Meier method. We found that breast cancer patients with high Tiam1 expression had lower DFS and 10-year OS (*P* = 0.003, respectively) rates than those with low Tiam1 expression (Fig. 4). Among the 153 breast cancer patients, 84 were early-stage and 69 were late-stage. In early-stage breast cancers, patients with low-level Tiam1 expression had higher DFS and OS rates compared with those with high-level Tiam1 expression (*P* = 0.021 and *P* = 0.043, respectively). Similarly, for patients with late-stage breast cancer, the expression status of Tiam1 protein was also correlated with DFS and 10-year OS rates (*P* = 0.003 and *P* = 0.007, respectively) (Fig. 5).

### Tiam1 overexpression is an independent prognostic biomarker in breast cancers by Cox proportional hazards regression model

Univariate analysis demonstrated that breast cancer patients with high Tiam1 expression had significantly lower DFS and 10-year OS rates than those with low Tiam1 expression tumors. Additionally, molecular type, clinical stage, and HER2 expression levels were also associated with DFS and 10-year OS rates, when Tiam1 was expressed (Table 3). These data suggested that Tiam1 could be used as a valuable prognostic factor in breast cancer. Therefore, multivariate analysis was performed using the Cox proportional hazards model for all of the significant variables, which were examined in the univariate survival analysis. We found that tumor stage proved to be a significant independent prognostic factor for DFS (HR: 0.567, 95 % CI: 0.413–0.778, *P* = 0.000) and 10-year OS (HR: 0.585, 95 % CI: 0.425–0.805, *P* = 0.001) in patients with breast cancers. Importantly, high Tiam1 expression also emerged as a significant independent prognostic factor for DFS (HR: 1.470, 95 % CI: 1.056–2.047, *P* = 0.022) and 10-year OS (HR: 1.549, 95 % CI: 1.112–2.157, *P* = 0.010) in patients with breast cancer (Table 3).

**Table 3:** Univariate and Multivariate survival analyses (Cox regression model) of various factors in 153 patients with breast cancer

Factors	DFS Hazard ratio (95 % CI)	*P* value	OS Hazard ratio (95 % CI)	*P* value
Univariate analyses				
Age	1.172(0.851–1.616)	0.331	1.162(0.844–1.601)	0.358
Menopausal status	1.015(0.831–1.239)	0.885	1.016(0.831–1.242)	0.878
Molecular type	1.373(0.995–1.896)	0.054	1.385(1.004–1.912)	0.047*
Tumor stage	0.685(0.590–0.796)	0.000**	0.694(0.599–0.805)	0.000**
LN metastasis	1.287(0.927–1.786)	0.131	1.335(0.961–1.855)	0.085
ER	1.171(0.848–1.616)	0.338	1.163(0.843–1.605)	0.359
PR	1.016(0.736–1.404)	0.922	1.048(0.760–1.446)	0.774
Her2	1.646(1.186–2.285)	0.003**	1.740(1.245–2.431)	0.001**
Ki-67	1.058(0.765–1.4622)	0.734	1.075(0.777–1.485)	0.663
Tiam1	1.781(1.276–2.487)	0.001**	1.671(1.204–2.320)	0.002**
Multivariate analyses				
Tiam1	1.470(1.056–2.047)	0.022*	1.549(1.112–2.157)	0.010*
Molecular type			1.340(0.963–1.865)	0.082
Tumor stage	0.567(0.413–0.778)	0.000**	0.585(0.425–0.805)	0.001**
Her2	0.585(0.290–1.179)	0.134	0.617(0.298–1.276)	0.617

*95 % CI* 95 % confidence interval

* *p* < 0.05, ** *p* < 0.01

## Discussion

Tiam1 was first detected as an invasion and metastasis gene by Habets et al. [5] and was identified as the specific GEF for the Rho-like GTPase. It was reported to participate in tumor progression and metastasis by activating Rho-like GTPases, specifically the Tiam1-Rac pathway [6, 7]. The small GTPases of the Rho family have been recognized as key regulators of signal transduction pathways that regulate the phenotypic cytoskeleton changes required for cell spreading, chemotaxis, and invasion [14]. Rho-like GTPases, in conjunction with GEFs, act as molecular switches by cycling between an active GTP-bound state and an inactive GDP-bound state. Michiels et al. [15] indicated that the Tiam1-Rac signaling pathway could be involved in the invasion and metastasis of cancer cells. Furthermore, the Tiam1-Rac signaling axis is able to cross-talk with other signaling pathways, such as the c-Jun N-terminal kinase (p38 MAPK) pathway and the extracellular signal-regulated kinase pathway [16, 17], which may implicate the Tiam1-Rac pathway in the regulation of gene transcription. Additionally, several studies reported the role of Tiam1 in the promotion of malignant transformation, tumor proliferation, invasion and metastasis [18–23]. Moreover, Tiam1 has been reported to participate in the regulation of the tumor microenvironment [24] by promoting the formation of E-cadherin-mediated cell-cell adhesion.

Accumulating evidence has shown that Tiam1 is expressed at relatively high levels in a variety of human malignancies, and a correlation between Tiam1 overexpression and several human cancers has been identified. Engers et al. [10] reported that Tiam1 expression was strongly increased in prostate cancer and high-grade prostate intraepithelium neoplasia (HG-PIN) lesions, compared with adjacent benign epithelium. The authors further demonstrated that Tiam1 overexpression in prostate cancer might be an independent prognostic marker of tumor recurrence. Similarly, Guo et al. [25] also indicated that the Tiam1-Rac1 axis is involved in pancreatic cancer cell proliferation and invasion. Inspired by these findings, we performed IHC staining of Tiam1 protein in 153 breast cancer samples, 67 DCIS and 63 adjacent non-tumor breast tissues. From our IHC results we found that Tiam1 is predominately localized in the cytoplasm, although some nuclear staining was also observed. These observations were consistent with the IF staining results in MDA-MB-231 breast cancer cells. Adam et al. [14] reported that Tiam1 is mainly located in the cytoplasm, and when cells are treated with heregulin-β1Tiam1 is detected in the nucleus. Otsuki et al. [26] also indicated that the expression of Tiam1 was demonstrated in the nuclei of cerebral neuronal cells. These findings are consistent with our observations. We also observed that Tiam1 protein is frequently overexpressed both in breast cancer and DCIS specimens, at significantly higher levels than normal breast tissues. DCIS is non-invasive breast cancer, which can increase the possibility of developing an invasive breast cancer. These results demonstrated that up-regulation of Tiam1 expression may occur at an early stage in breast cancer. Consistent with many other reports, Tiam1 may play an important role in tumorigenesis and malignant progression of breast cancer.

According to the molecular gene expression profiles of breast cancer biopsies, patients are classified into the following subtypes: ER positive (Luminal A and B) and ER negative (triple negative and HER2 positive) groups. Classification into the different subtypes can change tumor prognosis and responses to therapy. In addition, HER2 positive tumors are traditionally associated with poor prognosis. In fact, Yan et al. [27] also reported that in addition to ER status, HER2 status might play an important role in prognosis prediction. In our study, the expression of Tiam1 protein was significantly higher in breast cancers with low HER2 expression, Tiam1 expression was inverselyrelated to the HER2 expression. Multivariate survival analysis showed that Tiam1 expression level was a significantly and independent prognostic factor along with tumor stage in breast cancer, but not HER2 expression. It might be known that, Tiam1 could be involved in the tumorigenesis and tumor growth through different signal transduction pathway, such as Tiam1-Rac signaling pathway [15]. Wang et al. [28] reported that HER2 can activate Rac1 by activating PI3K/Akt pathway. Consequently, the molecular mechanism of Tiam1-regulation in breast cancer needs the further study to clarify.

As many studies demonstrated that, lymph node metastasis, as well as Her-2 and Ki-67 expression was the independent prognostic factors in breast cancer. Chen et al. [29] reported that Ki-67 expression was associated with lymph node metastasis frequently in all types of breast cancer. However, our contingency table analysis showed that Tiam1 expression did not correlate with lymph node metastasis, but associated with Ki-67 expression. Yang et al. [19] found that expression of Tiam1 in HCC tissues was associated with tumor stage, high AFP level and intrahepatic metastasis. Consistently, our study showed that up-regulation of Tiam1 was correlated with tumor stage in breast cancer. These results indicated that up-regulation of Tiam1 expression might promote the malignant potential of breast cancer. Thus, Tiam1 may act as a valuable molecular marker for breast cancer invasion, and play a key role in prognosis prediction.

In regards to survival, we found that high expression of Tiam1 protein was strongly associated with molecular subtype and tumor stage, which were important features associated with poor prognosis in breast cancer. Also, we found that Tiam1 expression was strongly related to survival rates in early- and late-stage tumors, and survival rates were significantly higher in patients with low Tiam1 expression than those with high Tiam1 expression. Consistent with these results, Yang et al. [9] reported that, in hepatocellular cancers (HCCs), Tiam1 expression was associated with shorter recurrence-free survival and disease-specific survival. In addition, Ding et al. [30] demonstrated that Tiam1 overexpression acts as an independent prognosis factor for HCC patients. Similarly, Hsueh et al. [31] reported that Tiam1 expression was associated with the clinicopathologic features and prognostic significance in patients with papillary thyroid carcinoma. Thus, increased expression of Tiam1 might promote the malignant potential of breast cancer, and Tiam1 overexpression might be used as a potential predictive biomarker of poor prognosis in patients with breast cancer.

Currently, many studies have reported that Tiam1 participates in several signaling pathways in tumor cells, and plays important roles in cancer progression, metastasis and invasion. Malliri et al. [32] reported that Tiam1 was essential for the formation, as well as the maintenance, of cadherin-based adhesions. Walch et al. [33] reported that Tiam1 was a regulator of E-cadherin-mediated cell adhesion, and indicated that in combination with E-cadherin expression, Tiam1 acts as a useful marker in gastric carcinogenesis. Hordijk et al. [34] and Liu et al. [35] reported that Tiam1 regulates the formation of cell-cell adhesion, induces Mesenchymal-Epithelial transition (MET), and thus inhibits epithelial cell migration. In part, this may be due to Tiam1’s ability to regulate E-cadherin expression, since loss of E-cadherin expression is considered to be a signature event in Epithelial-Mesenchymal transition (EMT), which is the reverse process of MET and has a pivotal role in cancer invasion and metastasis. Specifically, EMT is a process by which epithelial cells lose cell-cell adhesion, and gain migratory and invasive properties to become mesenchymal-like cells. Therefore, these data suggest that altered Tiam1 expression patterns might regulate a certain signaling pathway to play a key role in cancer invasion and metastasis. Further investigation is required to determine the role of Tiam1 in breast cancer progression.

## Conclusions

In conclusion, Tiam1 expression is frequently up-regulated in breast cancer, and Tiam1 overexpression correlated with clinicopathological parameters. Tiam1 may serve as a useful prognostic biomarker and a potential therapeutic target for patients with breast cancer.

## Abbreviations

Tiam1: T lymphoma invasion and metastasis 1
DCIS: Ductal carcinoma in situ
ER: Estrogen receptor
PR: Progesterone receptors
GEFs: Guanine nucleotide exchange factors
EMT: Epithelial-mesenchymal transition
MET: Mesenchymal-epithelial transition
